# Complete Genome Sequence of an Uncultivated Freshwater Bacteroidota Lineage

**DOI:** 10.1128/mra.00766-22

**Published:** 2022-09-28

**Authors:** Emanuel Mauch, Lucas Serra Moncadas, Adrian-Stefan Andrei

**Affiliations:** a Faculty of Science, University of Zurich, Zurich, Switzerland; b Microbial Evogenomics Lab, Limnological Station, Department of Plant and Microbial Biology, University of Zurich, Kilchberg, Switzerland; University of Delaware

## Abstract

We report here a complete metagenome-assembled genome belonging to the AKYH767 order within the Bacteroidota phylum. The recovered genome stems from a nonaxenic Amoebozoa culture from Lake Zürich and was assembled as a circular element at a length of 4.1 Mbp and a coverage of 115×.

## ANNOUNCEMENT

Bacteroidota phylum members have colonized a large spectrum of ecological niches, including soil, freshwater, marine and animal-associated, displaying rich metabolic potential. Bacteroidota emerged as a major freshwater bacterial lineage in 16S rRNA sequences analyses recovered from diverse aquatic habitats, playing a crucial role in degrading complex organic matter (1).

Unidentified Amoebozoa was nonaxenically cultured from a Lake Zürich (47° 13′ 20.9994″N, 8° 45′ 9.72″E, Swiss Confederation) sample. The cultures were maintained in tissue culture flasks filled with 50 mL of autoclaved Swiss Alpina water (Pearlwater, Termen, Switzerland). Planktothrix rubescens strain A7 served as food source. The DNA of this culture was extracted using the MagAttract HMW DNA kit (Qiagen, Hilden, Germany). It was further purified with Beckman Coulter AMPure XP magnetic beads and subsequently used for metagenomic sequencing on a Nanopore PromethION platform using a FLO-PRO002 (R9.4.1) flow cell. The sequencing 1D library was constructed with the SQK-LSK110 Ligation Sequencing kit (ONT, Oxford, UK) in conformity with manufacturer's instructions without any prior DNA fragmentation or size selection.

Obtained reads (approx. 2 million reads; mean read length 9 kbp, median read quality 11.7, *N*_50_ 14.4 kbp) were basecalled and quality trimmed (Q score 7) with Guppy 5.1.15 prior to assembly with Flye 2.9-b1768 (2). Potential contamination of the recovered circular chromosome (4 155 722 bp, coverage 115×) was assessed by CheckM v1.1.3 (94.89% complete, 0.63% contaminated) (3). Taxonomical classification was performed with GTDB-Tk v1.4.0 (4) and by comparing its 16S rRNA, predicted by barrnap 0.9, against SILVA database v138 (5). Prokka 1.12 (6) and NCBI’s PGAP pipeline were used for gene prediction and functional annotation. The assignment of KO identifiers to orthologous genes was performed by BlastKOALA (7). Pathway reconstruction was accomplished with the online KEGG Mapper-Reconstruction tool using the obtained KO numbers. PFAM domains were predicted using the pfam_scan.pl script with the PFAM database release 32 (8). gRodon R package (9) was applied to predict growth rates, while defense mechanisms diversity was explored with DefenseFinder (10). Default parameters were used except where otherwise specified.

GTDB-tk classified the obtained metagenome-assembled genome as belonging to an uncultivated order (AKYH767) within the Bacteroidota phylum. However, SILVA classification pointed toward Sphingobacteriales (88.45% identity) order within the same phylum. With a GC of 40.07%, the recovered metagenome-assembled genome exhibited four 869 CDS, 70 encoded transporters, and one rRNA operon. Genome-inferred metabolic reconstruction suggested a Gram-negative bacterium with fast duplication time (approx. 2.6 h) and an aerobic heterotrophic lifestyle. The circular chromosome (strain JAD_PAG50586_3) had the metabolic potential of synthesizing 50% of all proteinogenic amino acid as well as to degrade 60% of them. Anabolic (i.e., Gluconeogenesis, fatty acid synthesis) and catabolic (i.e., TCA-cycle, pyruvate oxidation, fatty acids degradation) metabolisms were found complete. Interestingly, the recovered genome displayed seven different defense mechanisms. Gliding motility was inferred based on the presence of 11 key genes.

### Data availability.

All sequence data are available through the National Center for Biotechnology Information (NCBI) via the BioProject PRJNA824509 (CP096135.1; GCA_023213195.1; SRX14779367). Additional proteome annotations (KEGG, Prokka and Pfam) are available in figshare repository: 10.6084/m9.figshare.20318880.
